# Liver tissue changes during and post 6-month spaceflight as measured by ultrasound radio frequency signal processing

**DOI:** 10.3389/fphys.2024.1460131

**Published:** 2024-09-30

**Authors:** Philippe Arbeille, Kathryn Zuj, Laurent Guillon

**Affiliations:** UMPS-CERCOM (Space Physiology and Medicine) School of Medicine University of Tours, Tours, France

**Keywords:** liver, radio frequency signal, RF, echography, spaceflight

## Abstract

**Background:**

Analysis of ultrasound radio frequency (RF) signals allows for the determination of the index of reflectivity (IR), which is a new measure that is dependent on tissue properties. Previous work has shown differences in the IR of the carotid artery wall with long-duration spaceflight; therefore, it was hypothesized that liver tissue would also show differences in this measure with spaceflight.

**Methods:**

The RF signal of a liver tissue region of interest (ROI) was displayed and processed along six different lines covering a surface of approximately 2 cm × 2 cm. The IR was calculated as the energy backscattered by the liver ROI divided by the total energy returned to the ultrasound probe.

**Results:**

Seven astronauts were investigated preflight, inflight on day 150, and postflight 4 days and 6 months after rerunning to Earth. Compared to preflight (63% ± 18%), the liver tissue ROI IR was significantly lower on flight day 150 (46% ± 14%; p = 0.027) and 4 days postflight (46% ± 19%; p = 0.025). At 6 months postflight, the IR returned to preflight values (59% ± 13%; p = 0.919).

**Conclusion:**

The significant decrease in the coefficient of reflectivity inflight and 4 days postflight indicates an alteration in the liver tissue that reduces the reflection of ultrasound waves. This change in tissue properties could either be due to the addition of particles that do not reflect ultrasound waves or structural or cellular changes that alter the reflectivity of the tissue.

## Introduction

Previous studies have shown a connection between insulin resistance and liver steatosis (Genazzani et al., 2024; Anousin et al., 2024). Elevation of an insulin resistance index has also been found after 6 months of spaceflight (Hughson et al., 2016). This suggests that liver structure may also be altered with long-duration spaceflight on the International Space Station (ISS).

Measurement of the index of reflectivity (IR), determined from analysis of the ultrasound radio frequency (RF) signal, has shown changes in the carotid artery wall with long-duration spaceflight (Arbeille et al., 2021). As this new ultrasound measurement was able to detect changes in tissue properties of the arterial wall, it is possible that similar measures of liver tissue will also identify changes in liver tissue properties with long-duration spaceflight on the ISS. Therefore, the objective of the present research was to determine whether the IR, determined by ultrasound RF signal processing, was altered for liver tissue during long-duration spaceflight and postflight recovery.

**FIGURE 1:**
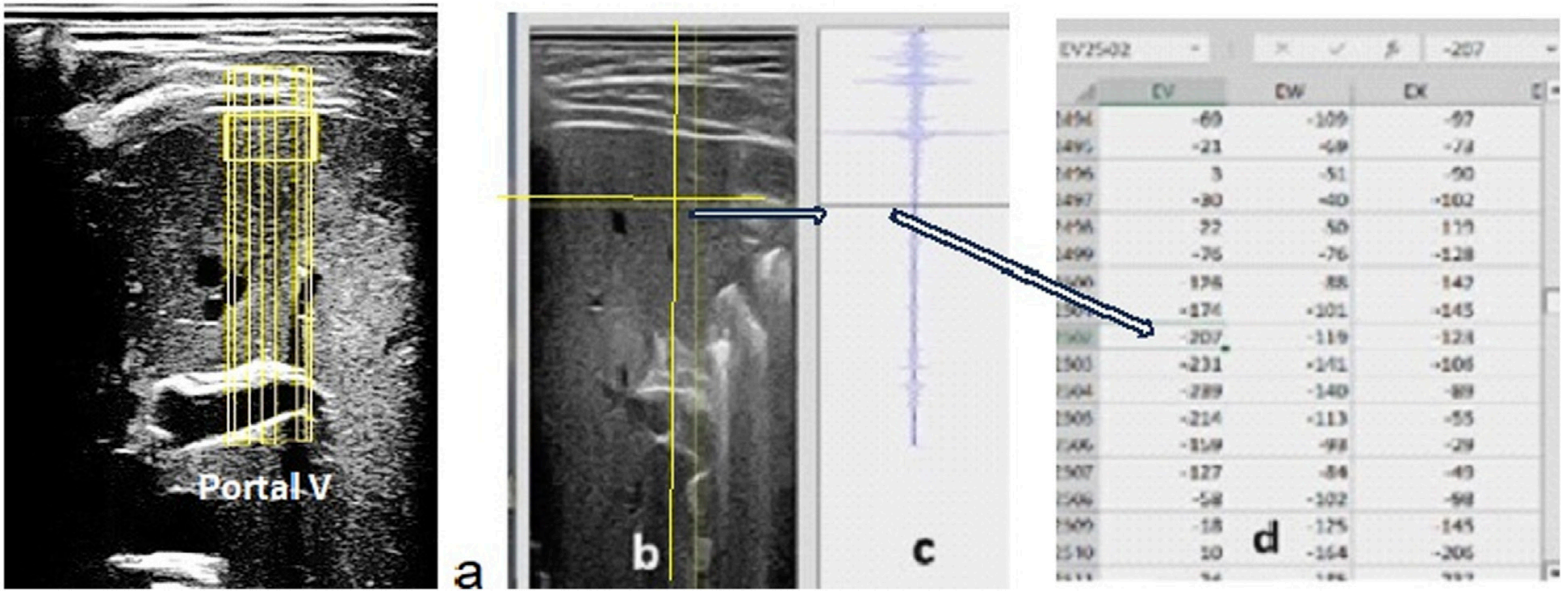
**(A)** B-mode liver view with the portal vein as a reference at the bottom of the image. The RF data are collected over the entire image but processed only along the six vertical lines passing through the liver region of interest. **(B)** Vertical and horizontal lines that the sonographer moves to determine the value of the energy reflected by the point at the intersection of these lines. Using this tool, the operator can identify the right and left and upper and lower limits of the ROI values and sum the values of the energy reflected along each vertical segment of the ROI on the six lines selected in the data table. **(C)** The RF signal presented corresponds to one full vertical ultrasound line crossing the sample. **(D)** The tabulated data display the values of the amplitude of the ultrasound signal reflected at any point in the image. The square of the amplitude values (minus the background noise estimated at the bottom of the ultrasound image) was used to estimate the energy reflected at each point.

The RF signal is the native ultrasound signal that is processed by the ultrasound system manufacturer’s software (i.e., filtered, smoothed, and amplified) to construct B-mode (black and white) ultrasound images. During operation, ultrasound probes emit hundreds of ultrasound beams in the plane below the probe head. As the beams reflect off successive interfaces along linear paths, changes in the reflected waves are captured by the probe as the radio frequency signal (RF) (Figure 1C).

The native RF signal is recognized in ultrasound science with applications designed to accurately track tissue displacement in the carotid artery walls (Palombo et al., 2012) or brain tissue (Jurkonis et al., 2020) and to study tissue characteristics. Tissue microstructure has been examined (Maleke and Konofagou, 2008), as have changes in tissue properties after being heated by focused ultrasound, with reports of a relationship between an increased stiffness and the reduction in micro-displacement inside the tissue (Maleke and Konofagou, 2008). RF recording and processing were also used in studies of carotid artery stenosis to evaluate the average blood integrated-back scattered energy within the lumen at proximal and distal segments to gain an estimate of a functional ischemia parameter (Okada et al., 2019). At the abdominal level, the processing of the ultrasound image content or the RF signal was applied to identify tissue aspect changes such as liver steatosis or fibrosis (Byeong Geun et al., 2024; Moradi et al., 2006) and detection/characterization of prostate malignant tumors (Schmitz et al., 1999). Lastly, RF signal processing has been used to investigate membranes of tumors of the inner eye (Trier, 1983).

The present study was based on the use of the index of reflectivity (IR), as defined previously for the evaluation of carotid wall changes during spaceflight and dry immersion (Arbeille et al., 2021; 2022). Using the same method, the RF signal of a liver tissue region of interest (ROI) was collected and analyzed preflight, inflight, and postflight, and the IRs of these ROIs were calculated. We hypothesized that the morphological and/or mechanical properties of the liver tissue structure and or content would be affected by spaceflight and detected as a change in the corresponding IR.

## Research design and methods

### Population investigated

Approximately 1 month before a 6-month spaceflight, after 150 days of flight, and 4 days and 6 months after return to Earth, seven astronauts (five men and two women; age: 44 ± 3 years; height: 177 ± 5 cm; weight: 76 ± 11 kg) underwent an ultrasound investigation where liver B-mode images and RF signals were acquired. The protocol was approved by the University of Waterloo Office of Research Ethics, the Johnson Space Center Committee for the Protection of Human Subjects, the NASA Human Research Medical Review Board, the European Space Agency Medical Review Board, and the Japanese Space Agency Research Ethics Board (Study Protocol #Pro1222; NASA MPA116301606HR; FWA00019876) in accordance with the Declaration of Helsinki. Each participant was informed in detail about the experiment and gave informed consent before participating.

### Data acquisition

A 2D, B-mode ultrasound image of the liver with the portal vein at the bottom of the image was acquired, and the RF signal of the entire image was recorded. The RF signal was displayed and processed along each ultrasound line selected by the sonographer (Figure 1). The RF signal was processed along six different lines covering a liver ROI of approximately 2 cm × 2 cm on the 2D image of the liver, close to the upper limit of the right lobe. The index of reflectivity was calculated from the RF signal as the quotient of the energy back scattered by the liver tissue ROI along each of the six vertical RF lines and the total energy returned to the ultrasound probe along the same selected lines. Therefore, the index of reflectivity represented the percentage of the total energy returned along the selected line by the liver ROI to the ultrasound probe.

The IR of one ROI segment (IRseg) was expressed as the sum of the energy reflected by this vertical segment (Eseg) divided by the sum of the total energy reflected along the line (Etot) minus the sum of the energy reflected by the portion of the line between the upper limit of the segment and the skin (Esup) with IRseg = Eseg/(Etot − Esup). The IR value (expressed in %) for each sample was the average value of the six IRseg calculated along the six segments inside the liver ROI. Caution was taken with the selection of the ultrasound view from which the RF signal was processed to optimize the reproducibility and accuracy of the ultrasound measurements. During spaceflight, the astronauts were guided (vocally) from the ground to locate the ultrasound probe on the portal vein acoustic window. The trained sonographer on the ground then teleoperated the ultrasound probe sensor orientation (tilt and rotation) to obtain a clear image of the portal vein. The radiofrequency signal capture (internal software) was then activated, and the recording was stored on the ISS device hard disc for later downlink.

The index of reflectivity was calculated on an ROI located at the upper part of the liver’s right lobe on an image showing the portal vein trunk at the bottom. This condition was required to confirm that the liver ROI was in the same area for each of the imaging sessions. Additionally, as the accuracy of any ultrasound measurement depends mostly on the quality of the B-mode image and that image quality is partially operator-dependent, the same two operators performed the assessments pre, during, and postflight.

The astronauts remained supine during the pre and postflight assessments and were free floating inside the ISS for the inflight measurement. The same ultrasound device (Sonoscanner, Paris, France) with the same probe (curved array 3.5 Mhz) and ultrasound presets (frequency, depth, and gain) was used for each data collection.

Each index of reflectivity value was a mean of six IR segments placed on the B-mode image. For one crew member, only one RF line was available for one of the images, and it was still included in the analysis. As the ultrasound was teleoperated, the time to find the appropriate liver view and the RF signal on astronauts on board the ISS did not exceed 2 min.

### Statistical analysis

The effects of spaceflight were determined using a one-way, repeated measures analysis of variance (SigmaPlot, 12.5; Systat Software, San Jose, CA) with Tukey *post hoc* testing performed to assess all pairwise comparisons. Significance was set at p< 0.05, with all results reported as mean ± SD.

Our ROI size is based on our previous work on reflectance changes with fluid shift analogs on seven subjects exposed to microgravity for 6 months and 12 people exposed to dry immersion for 4 days (Arbeille et al., 2021; 2022), indicating a large effect (d = 1.3) to increase reflectance. Based on a one-tailed dependent t-test of alpha = 0.05, n = 5 participants is sufficient to have 80% power to detect differences over time (G*Power v 3.1.9.7).

## Results

Ultrasound images of the liver with the portal vein at the bottom of the image as a landmark to confirm the upper part of the right liver lobe were successfully recorded for all astronauts. The RF signal over the entire part of this image was recorded during spaceflight and downlinked to the ground for processing. Processing on the ground consisted of displaying the RF data for the entire B-mode image (tabulated data) and then moving two lines on the B-mode image to identify the liver tissue ROI to be investigated. The data columns corresponding to the sample segment limits were identified, and the different segment energy values were calculated (Figure 1). The liver RF data were collected for seven astronauts preflight, inflight (day 150), and postflight 4 days (R + 4) and 6 months after returning to Earth.

**FIGURE 2:**
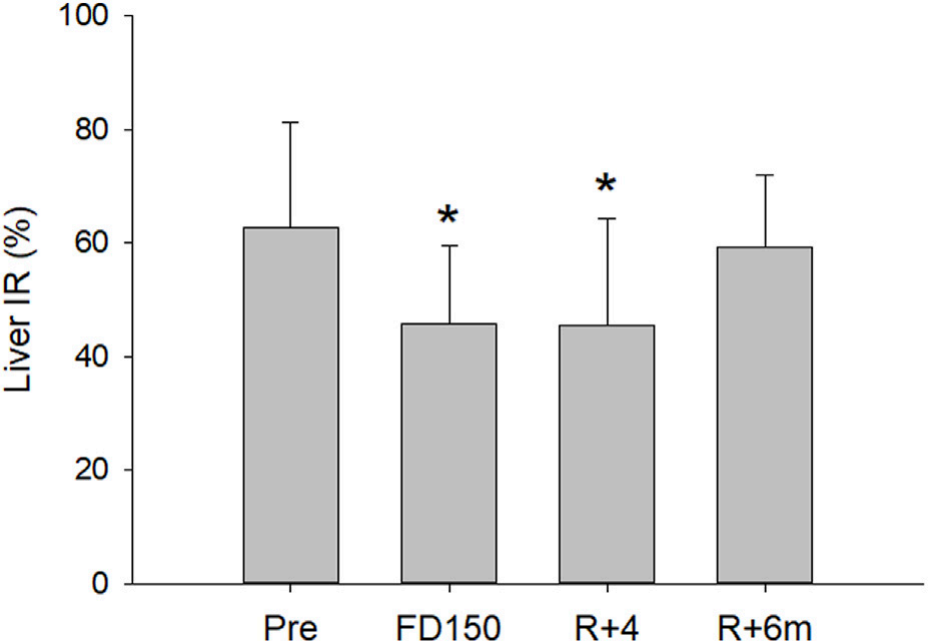
Mean (±SD) liver IR values preflight (pre), after 150 days of spaceflight (FD150), 4 days after returning to Earth (R+4), and 6 months after returning to Earth (R+6 m). The liver IR was decreased both inflight (FD150) and postflight (R+4), while at R+6 m, the value returned to preflight levels (statistically different from Pre indicated by *, p < 0.05)

**FIGURE 3:**
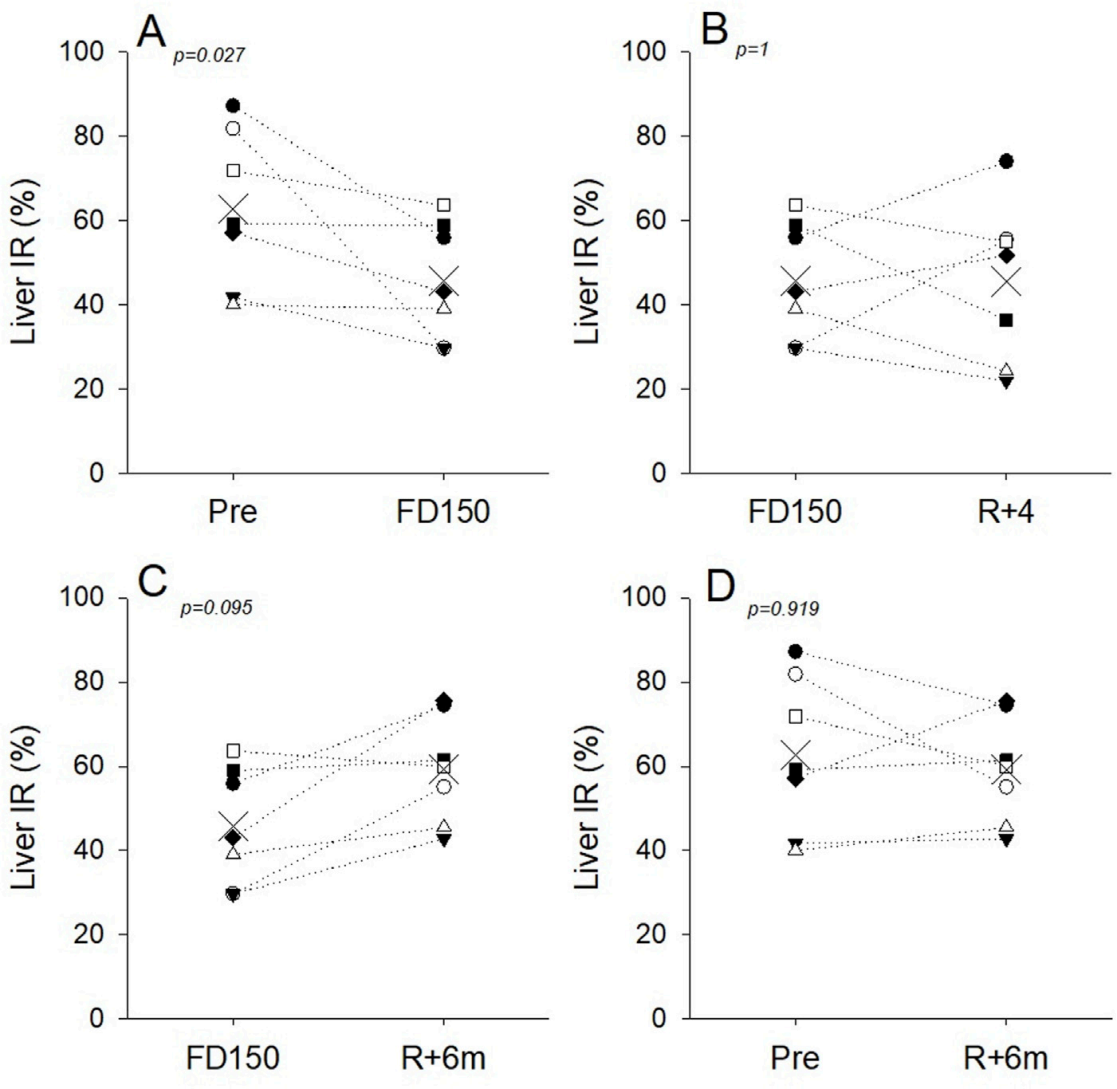
Individual liver IR values and group means (X symbol) preflight (Pre), during spaceflight (FD150), 4 days (R+4), and 6 months (R+6m) after returning to Earth. Dotted lines are included to visualize individual responses and do not indicate a linear time course. **(A)** Liver IR was decreased in all astronauts on FD150 compared to pre; **(B)** at R+4, IR tended to increase in three astronauts and decreased further in the other four; **(C)** IR tended to recover in five of the seven astronauts at R+6m; **(D)** at 6 months, postflight IR has recovered to preflight values for five of the seven astronauts.

The analysis found a significant effect of time on the IR (p = 0.007). Compared to preflight levels (63% ± 18%), the IR of the liver tissue ROI was significantly lower inflight after 150 days (46% ± 15%; p = 0.022) and on day R + 4 postflight (46% ± 19%; p = 0.023), with no difference found between flight day 150 and R+4 (*p* = 0.999). Assessment of the liver IR 6 months postflight found that IR had returned to preflight levels (59% ± 13%; p = 0.941) (Figures 2, 3).

## Discussion

The current study utilized analysis of the ultrasound radio frequency signal to evaluate liver tissue properties during and after long-duration spaceflight. After 150 days of spaceflight, the IR of the liver was found to be significantly reduced, suggesting alterations in tissue content or structure with spaceflight. This study confirmed that the RF signal processing technique to assess IR provides an additional, noninvasive measure for assessing adaptations to spaceflight.

The ultrasound B-mode images from which the RF signal was recorded during spaceflight were of high quality, as the probe transducer orientation and device setting were successfully teleoperated (controlled) in real-time by an expert sonographer in the ground space center. The teleoperation mode allowed the sonographer on the ground to optimize the view of the liver and trigger the RF capture at the most appropriate moment. Additionally, the expert evaluated the echo image and the corresponding RF traces recorded and could repeat the capture if necessary to acquire the best RF data. It was not possible to verify that the ultrasound probe’s position over the portal vein acoustic window during spaceflight was exactly the same as it was on the ground. However, the astronauts were able to locate this acoustic window easily from the basic anatomical references used on the ground. Additionally, even if the liver had moved by 1 cm, changed its volume from fluid shifts, or was structurally modified, the astronaut and the sonographer on the ground found the same portal vein image inflight and on the ground, indicating that the RF signal was collected on the same liver area (ROI) during spaceflight as on the ground.

The alteration in the index of reflectivity measured inside the liver tissue suggests changes in the physical properties of the tissue in response to spaceflight. While these changes persisted 4 days after returning to Earth, the index returned to preflight values after 6 months. The decrease in the index of reflectivity means that the liver tissue reflected less of the ultrasound waves during flight than preflight. A reduction in liver IR could be due to particles that do not reflect ultrasound entering this organ (change in water/tissue composition including fat content), potential structural or cellular changes (more collagen and more glycation in the organ), higher superficial tissue absorption, or any combination of these three processes.

During spaceflight, the decrease in the index of reflectivity could be related to fluid shifts toward the upper part of the body. Exposure to microgravity shifts fluid toward the head and neck with potential storage as increased blood pooling in the neck and abdominal vessels and organs, as indicated by the increase in jugular and portal vein volume during spaceflight (Arbeille et al., 2015). Returning to Earth’s surface, fluid shifts away from the head and neck with rapid recovery of blood vessels. However, with the decreased liver IR found to persist 4 days post-flight, recovery of the liver tissue liquid content may take much longer. On the other hand, the decrease in the index of reflectivity may also be the result of structural changes inside the organ, which would require more than several days to recover and should also be considered a possible second process not related to the fluid shifts.

Individual variability was also noted in IR measurements. While on average, liver IR values were not different on FD150 and R+4, values for four astronauts were lower on R+4 than on FD150, and values for the other three astronauts had increased toward preflight levels on R+4. (Figure 3B). The hypothesis of liver tissue structural modifications is supported by the fact that the decrease in IR was still present in four of the seven astronauts investigated at R+4 and in two of the seven astronauts after 6 months (Figures 3C, D). Conversely, for the other three astronauts who recovered at R+4, changes in liver IR with spaceflight may be primarily due to inflight fluid shifts that may disappear during the 4 first days postflight. In the absence of histological confirmation, both processes must be considered potentially contributing to the observed liver IR changes.

Changes in liver position or morphology may also contribute to the altered liver IR with spaceflight. Microgravity may result in changes in liver position, or liver tissue may be compressed with increased thoracic or abdominal pressure with spaceflight. However, the hypothesis of possible liver compression by surrounding organs or in relation to fluid shifts does not look likely as there was no difference in the density of the hepatic vein (subjectively evaluated) between pre and inflight ultrasound images. Increased portal vein size with spaceflight does indicate fluid retention and blood pooling at the level of the liver, but it is unknown if this has a compression effect on liver tissue.

The index of reflectivity provides an easily acquired, noninvasive index characterizing changes in tissue properties with spaceflight. Several other studies used the RF signal or the image brightness distribution to characterize the tissue structure or content of various organs. A liver ultrasound image scan showed signs of fat storage in the liver (steatosis), inflammation and swelling (hepatitis), and scar tissue (fibrosis or cirrhosis) (Byeong Geun et al., 2024). At the prostatic level, a method based on processing radio frequency ultrasonic echo signals in humans was used for the detection of prostatic carcinoma and estimating the probability of malignancy (Schmitz et al., 1999). Another RF-based study reported encouraging results on animal studies designed to analyze the capability of the fractal dimension of RF time series (AHDRFT) in characterizing, *in vitro*, different tissue types (Moradi et al., 2006; 2007). At the eye, a technique for radio frequency tissue characterization with a hand-held transducer in the 5–25 MHz frequency range was successfully tested on ocular tissues, especially on membranes and tumors of the inner eye (Tiers, 1983). Lastly, ultrasound-guided percutaneous RF ablation has been widely used for tumors (HCCs) and metastases in the liver and could improve overall and disease-free survival rates (Jin Woong et al., 2015).

However, none of the above studies used the very simple quantification of the RF signal energy backscattered from a volume of an organ as we do in the present study. The RF signal, being the native ultrasound signal, is present in every ultrasound machine but is not necessarily displayed. An option for this measurement can be installed on every ultrasound device with the appropriate software to process the RF data. Moreover, the RF signal collection is performed with a 2D, B-mode image, which is used to easily locate the tissue ROI to record the RF signal. The RF signal of the entire image was recorded in the ultrasound device used for the current study, allowing the sonographer to choose any sample area in the image, and the corresponding tabulated data of the tissue sample was immediately displayed.

The analysis of the RF signal in different areas of organs provides an additional metric to conventional parameters based on the quantification of the ultrasound image brightness. In many cases, assessment of the IR is more sensitive, as alterations in this variable have been found for images of the walls of the common carotid artery and superficial muscles despite traditional ultrasound image assessments finding limited or no changes in the carotid wall or neck muscles (Arbeille et al., 2021). Similar observations can also be made with the liver, as previous assessments have not identified any alterations in the organ despite the portal vein being increased in a majority of astronauts and liver IR reduced in the current study with similar conditions.

Nevertheless, in the absence of anatomical analysis of the organ structure investigated by optical or electron microscopy, we cannot identify the tissue- or cellular-level mechanism responsible for the change in the index of reflectivity, although the ultrasound image can be enough to suggest steatosis as a possible diagnostic clue. Presently, similar studies analyzing liver tissue by RF measures are being conducted using microgravity analogs, such as long-term bed rest, associated with MRI investigations. In the future, the presence of an MRI modality onboard the space station will probably help clarify this question.

## Conclusion

The liver index of reflectivity, as evaluated from the RF signal, provides additional information on the structure or content of the liver tissue beyond the traditional B-mode image. The results support the hypothesis of liver tissue remodeling, a change in liver tissue content, or liver positioning change or compression due to increased thoracic or abdominal pressure during and after spaceflight.

## Data Availability

The raw data supporting the conclusions of this article will be made available by the authors, without undue reservation.
